# Associations between cardiac adipose tissue and abdominal visceral fat and muscle based on computed tomography area and density

**DOI:** 10.1038/s41598-025-06167-7

**Published:** 2025-06-20

**Authors:** Babak Salam, Sebastian Nowak, Maike Theis, Alexander Böhner, Thomas M. Vollbrecht, Marilia B. Voigt, Christoph Endler, Tatjana Dell, Alexander Isaak, Claus C. Pieper, Daniel Kuetting, Alois M. Sprinkart, Julian A. Luetkens

**Affiliations:** 1 Department of Diagnostic and Interventional Radiology, University Hospital Bonn, Venusberg-Campus 1, 53127 Bonn, Germany; 2 Quantitative Imaging Lab Bonn (QILaB), Bonn, Germany

**Keywords:** Inflammaging, Frailty, Body composition, Computed tomography, Predictive markers, Prognostic markers

## Abstract

Computed Tomography (CT)-derived body composition parameters of cardiac adipose tissue (CAT), as well as abdominal adipose and muscle tissue are surrogates for the patient’s clinical condition and have prognostic implications. However, associations between the compositions of these diverse tissue compartments remain poorly investigated. This study aimed to investigate the associations between CT-derived parameters of CAT and abdominal adipose and muscle tissues. Retrospective analysis of CT scans from 842 patients was conducted, with measurements of CAT taken at the aortic valve level and abdominal tissues assessed at the L3/L4 intervertebral disc space. Area and density were calculated for each tissue compartment using single-slice images. Strong positive correlations were found between CAT area and visceral adipose tissue (VAT) area (*R* = .755, *P* < .001), as well as moderate correlations between CAT density and VAT density (*R* = .521, *P* < .001). Additionally, skeletal muscle (SM) area exhibited modest positive correlations with VAT area (*R* = .370, *P* < .001), CAT area (*R* = .300, *P* < .001), and SM density (*R* = .356, *P* < .001). No significant differences were observed between genders in the correlation strengths of these associations. These findings indicate a systematic pattern of body composition alterations, advocating for the inclusion of comprehensive body composition analysis in future studies and emphasizing the need for a deeper understanding of the underlying systemic processes influencing body composition.

## Introduction

Research interest continues to intensify concerning the predictive value of imaging biomarkers of body composition for cardiovascular and malignant disease, as well as for assessing intervention outcomes. Alterations in adipose and muscle tissue composition have proven to be objective surrogates for the patients general condition, influenced, among other factors, by the aging process^1–3^.

As the body ages, it undergoes chronic, systemic, low-grade proinflammatory processes, often referred to as “inflammaging”^4,5^. Adipose tissue, serving as the largest endocrine organ in humans, plays a pivotal role in age-related metabolic disorders and longevity^6^. Particularly, visceral abdominal adipose tissue (VAT) significantly contributes to the pathophysiology of cardiometabolic disease by increased secretion of adipokines, subsequently inducing a subclinical inflammatory state^7,8^. These inflammatory processes are not limited solely to VAT but are similarly observed in various fat compartments, including cardiac adipose tissue (CAT)^9^.

While chronic low-grade inflammation develops with aging, it is also apparent that age-related inflammatory conditions exacerbate the aging process. Multimorbidity contributes to an accumulation of deficits that participate in the onset and assessment of frailty. In this context, inflammaging is directly linked to an increased risk of age-related chronic diseases and frailty^10^. Sarcopenia, as part of the frailty syndrome, has been shown to correlate with poorer outcomes after cardiovascular and malignant disease as well as worsened outcomes following interventions^11–13^.

In this context, Computed Tomography (CT) offers an accurate method to opportunistically quantify body composition, yielding surrogate markers for the patient’s clinical condition.

While CT parameters of abdominal and pericardial fat, as well as abdominal muscle composition, have demonstrated prognostic implications in various diseases, associations between these body composition parameters across compartments are less well understood^1–3,8,14,15^. This study thus aimed to investigate associations between CT parameters of CAT and abdominal adipose and muscle tissue.

## Methods

### Study population

**Fig. 1:**
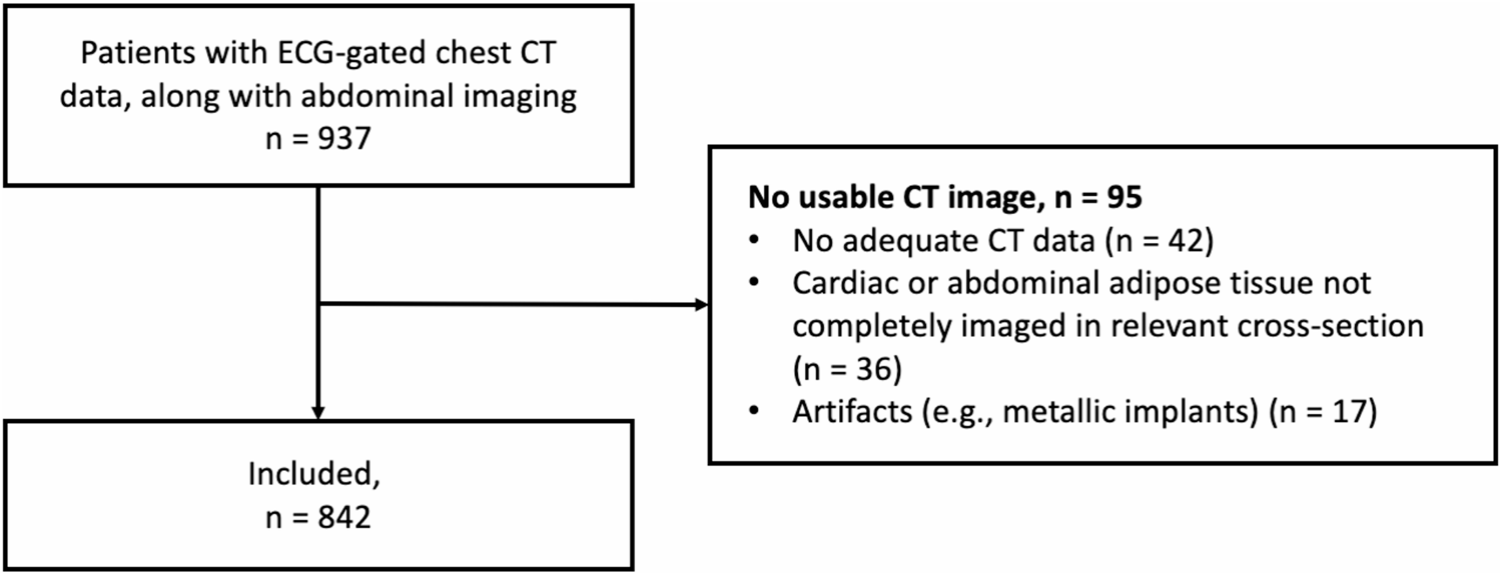
Flow diagram shows included patients with available Electrocardiogram (ECG)-gated chest CT data, along with abdominal imaging and patients who had to be excluded from analysis.

The study was approved by the Ethics Committee of the University of Bonn (Medical Faculty), with a waiver of written informed consent due to its retrospective design. All procedures adhered to the ethical standards of the 1964 Declaration of Helsinki and its later amendments. Electrocardiogram (ECG)-gated chest CT data, along with abdominal imaging, were retrospectively analyzed for 937 patients. Inclusion criteria required that scans covered both the aortic valve and the L3/L4 intervertebral disc space, which served as anatomical landmarks for body composition assessment^16,17^. After excluding patients with non-evaluable CT scans (e.g., due to artifacts), 842 patients remained for the final analysis (Fig. 1**)**.

## CT protocol and image acquisition

CT scans were performed on either a dual-source CT scanner (Somatom Force, Siemens Healthineers, Forchheim, Germany) or a single-source CT (Brilliance 64, Philips, Best, the Netherlands) with parameters set as follows: tube voltage of 120 kVp, automatic tube current modulation, and detector collimation of 2 × 192 × 0.6 mm or 64 × 0.625 mm. ECG-gated imaging of the heart (prospective gating at the dual-source CT, retrospective gating on the single-source system) was followed by a full abdominal scan during the arterial phase. Intravenous nonionic contrast agent (Iomeron, 300 mg/mL; Bracco-Eisai Co., Ltd.) was administered (70 mL at 5 mL/s), followed by a 30 mL saline chaser. Only arterial phase images were considered for body composition analysis, to prevent any potential bias due to the contrast phase. For each patient, anonymized single-slice images at the aortic valve (AV) and L3/L4 levels were retrieved from the local picture archiving and communication system (IMPAX, Dedalus HealthCare GmbH, Germany) for further analysis.

## Segmentation and quantification of cardiac adipose tissue

For CAT assessment, slices with a thickness of 1 mm acquired or reconstructed in the diastolic phase (67–90% of the RR interval) were analyzed to optimize pericardial visualization. CAT segmentation was performed in a way that allows distinguishing between epicardial adipose tissue (EAT), located between the myocardium and pericardium, from paracardial adipose tissue (PAT), situated on the outer surface of the pericardium^18,19^. Single-slice axial images at the level of the aortic valve were used, as these measurements have been shown to strongly correlate with total EAT and PAT volumes^17^.

**Fig. 2:**
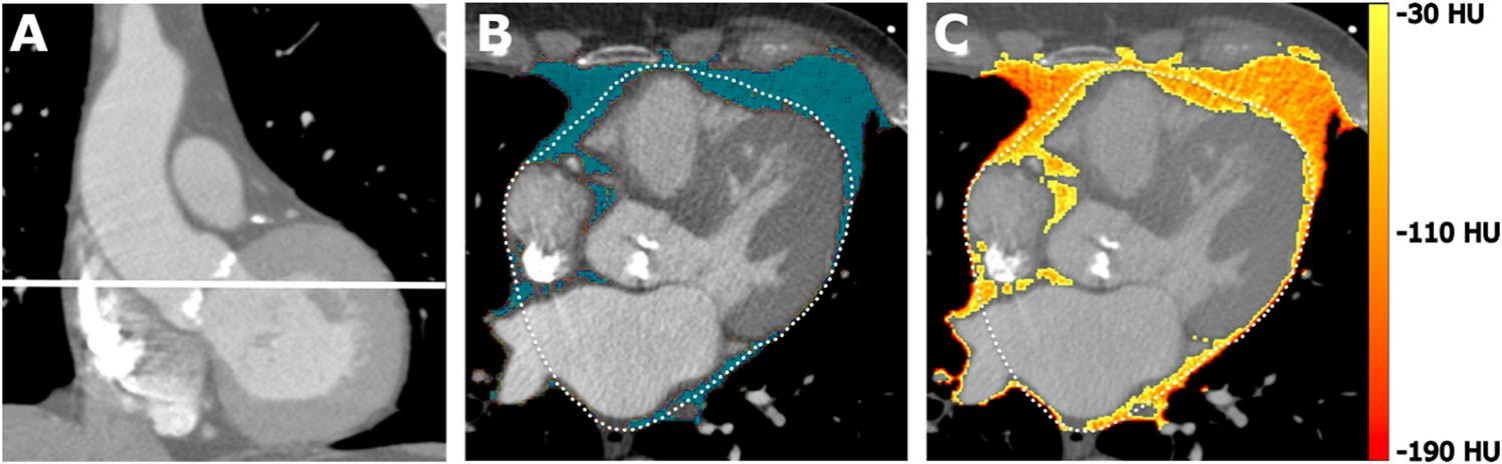
Synopsis of cardiac adipose tissue analysis. Single-slice cross sectional CT images at the level of the aortic valve were exported for analysis (**A**). Cardiac adipose tissue area was assessed within these cross-sections based on manual delineation of the pericardium and applying densitometric thresholds (**B**). Mean attenuation of the cardiac adipose tissue was calculated to obtain the inflammatory activity of the adipose tissue (**C**). HU = Hounsfield Units.

CAT analysis was performed using free open-source software (3D Slicer 4.11, Slicer.org)^20^. One experienced cardiovascular radiologist, who was blinded to the patients` clinical features, identified the anatomical landmark of the aortic valve and performed subsequent delineation of EAT and PAT at this level. Adipose tissue was identified by an attenuation threshold range of −190 to −30 Hounsfield units (HU)^3^. In two steps, first the adipose tissue area within the defined segments was quantified and secondly the mean adipose tissue density of the respective adipose tissue compartment was assessed (Fig. 2**)**. In this context, mean adipose tissue density was considered a surrogate marker for inflammatory activity, reflecting the shift from lipid to aqueous phase in inflamed adipose tissue^3,21^. To mitigate potential partial volume effects in density measurements, the outermost pixel layer of each adipose compartment was eroded.

## Segmentation and quantification of abdominal tissues

**Fig. 3:**
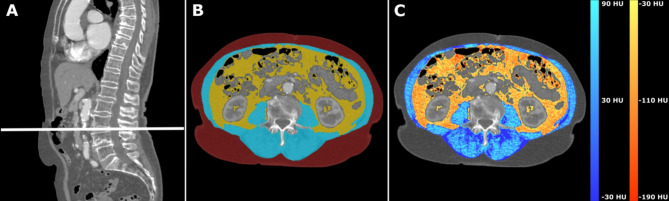
Synopsis of abdominal fat and muscle tissue analysis. Single-slice cross sectional CT images at the level of the intervertebral disc space L3/L4 were automatically identified for analysis (**A**). Subsequently, areas of visceral adipose tissue (yellow) were separated from subcutaneous adipose tissue (red) and abdominal skeletal muscle (blue) applying the validated Deep-Learning Pipeline (**B**). In addition to adipose tissue area, mean attenuations of visceral adipose tissue area, as well as skeletal muscle area and attenuation were calculated to determine overall inflammatory activity, respectively myosteatosis (**C**). HU = Hounsfield Units.

For abdominal tissue analysis, single-slice axial cross-sections at the L3/L4 intervertebral disc level were analyzed, as measurements at this level correlate strongly with total volumes of abdominal fat compartments (visceral adipose tissue, VAT; subcutaneous adipose tissue, SAT) and skeletal muscle (SM)^16^. An automated deep-learning pipeline segmented and quantified the SAT, VAT, and SM areas, with further calculation of mean density for SAT and VAT^22^. For SM analysis, the mean attenuation was calculated as a measure of myosteatosis (Fig. 3**)**. Again, as part of the density calculation, the outermost pixel layer of the SAT, VAT, and SM compartments was eroded.

### Statistical analysis

Statistical analysis was performed using SPSS Statistics 29 (IBM, Armonk, NY, USA). Patient characteristics are presented as mean ± standard deviation (SD) for continuous variables and counts and percentages for categorical variables. Correlation analysis was performed using Pearson correlation coefficient. Interpretation of correlation was defined as follows: almost perfect: >0.80, substantial: 0.61–0.80, moderate: 0.41–0.60, fair: 0.21–0.40, poor: 0.00–0.20)^23^. Student’s t-test was used to compare continuous variables between male and female groups. Dichotomous variables were compared using the χ2 test. Fisher’s r-to-Z transformation and Z-test were used to compare correlations of the same variables across sex (male vs. female), age (younger vs. older), and BMI (low vs. high) groups. To account for multiple testing, a Bonferroni correction was applied to all Pearson correlations. Additionally, multiple linear regression analyses were conducted, including age and BMI as covariates, to assess their influence on body composition parameters. The level of statistical significance was set to *P* <.05.

## Results

### Anthropometric and CT-derived characteristics of the study population

Out of the 842 patients finally included in the study, 421 (50.0%) were males. Mean age of the study population was 80.9 ± 6.2 years. The anthropometric parameters showed a mean height of 167.4 ± 9.3 cm, weight of 74.0 ± 14.9 kg, and BMI of 26.3 ± 4.6 kg/m². The CT-derived measurements revealed a mean CAT area of 20.3 ± 10.8 cm² and a CAT density of −86.1 ± 6.4 HU. VAT measurements yielded a mean area of 177.9 ± 107.0 cm² and a density of −84.0 ± 10.7 HU. Additionally, the mean SM area was 127.3 ± 29.1 cm² with a density of 23.3 ± 7.9 HU. A summary of all measured CT parameters is presented in Table 1.

**Table 1:** CT characteristics of the study population (*n* = 842).

Variable	Patient cohort (*n* = 842)
*CT characteristics*	
Visceral adipose tissue area (cm^2^)	177.9 ± 107.0
Visceral adipose tissue density (HU)	−84.0 ± 10.7
Subcutaneous adipose tissue area (cm^2^)	211.1 ± 102.3
Skeletal muscle tissue area (cm^2^)	127.3 ± 29.1
Skeletal muscle tissue density (HU)	23.3 ± 7.9
Cardiac adipose tissue area (cm^2^)	20.3 ± 10.8
Cardiac adipose tissue density (HU)	−86.1 ± 6.4
Epicardial adipose tissue area (cm^2^)	12.9 ± 6.3
Epicardial adipose tissue density (HU)	−81.2 ± 6.3
Paracardial adipose tissue area (cm^2^)	7.3 ± 5.9
Paracardial adipose tissue density (HU)	−94.5 ± 9.4

## Correlations among anthropometric and CT-derived characteristics

Substantial correlations were observed between anthropometric parameters (BMI and weight) and body composition metrics. BMI correlated moderately to strongly with VAT area (*r* =.630), CAT area (*r* =.515), and VAT density (*r* = -.523; *P* <.001 for all). Similarly, weight demonstrated strong correlations with VAT area (*r* =.708), CAT area (*r* =.602), and VAT density (*r* = -.487; all *P* <.001).

**Fig. 4:**
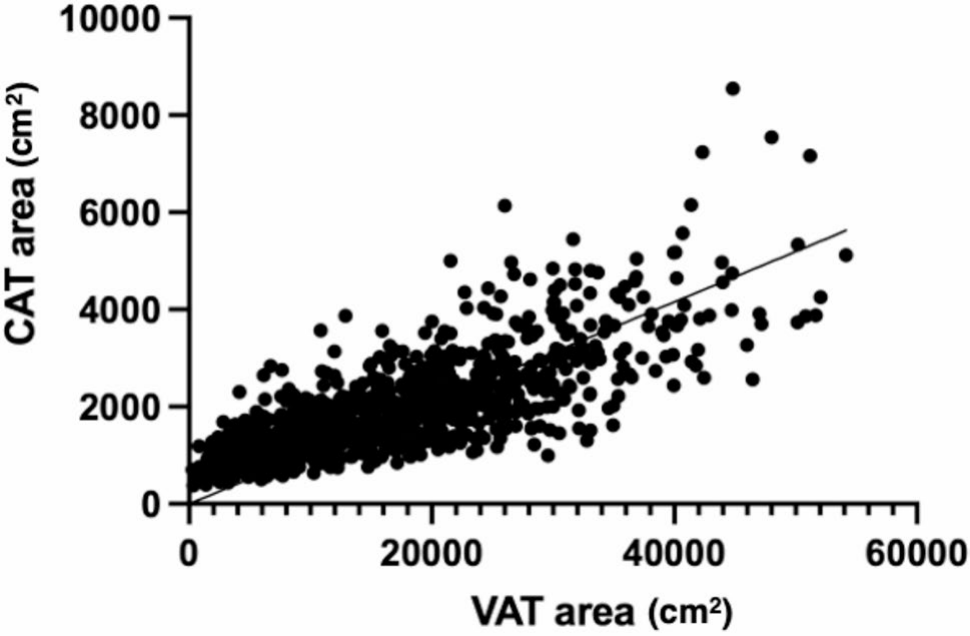
Scatter plot illustrating the association between cardiac adipose tissue (CAT) area and visceral adipose tissue (VAT) area.

Within adipose tissue compartments, VAT area exhibited substantial positive correlations with CAT area (*r* =.755, Fig. 4), EAT area (*r* =.663), and PAT area (*r* =.666; all *P* <.001). Moderate correlations were found between VAT density and CAT density (*r* =.521), PAT density (*r* =.505), and EAT density (*r* =.279; all *P* <.001). Strong negative correlations were identified between adipose tissue area and density values both within compartments (e.g., VAT area vs. VAT density: *r* = -.746, *P* <.001) and across compartments (e.g., CAT area vs. VAT density: *r* = -.549, *P* <.001).

**Fig. 5:**
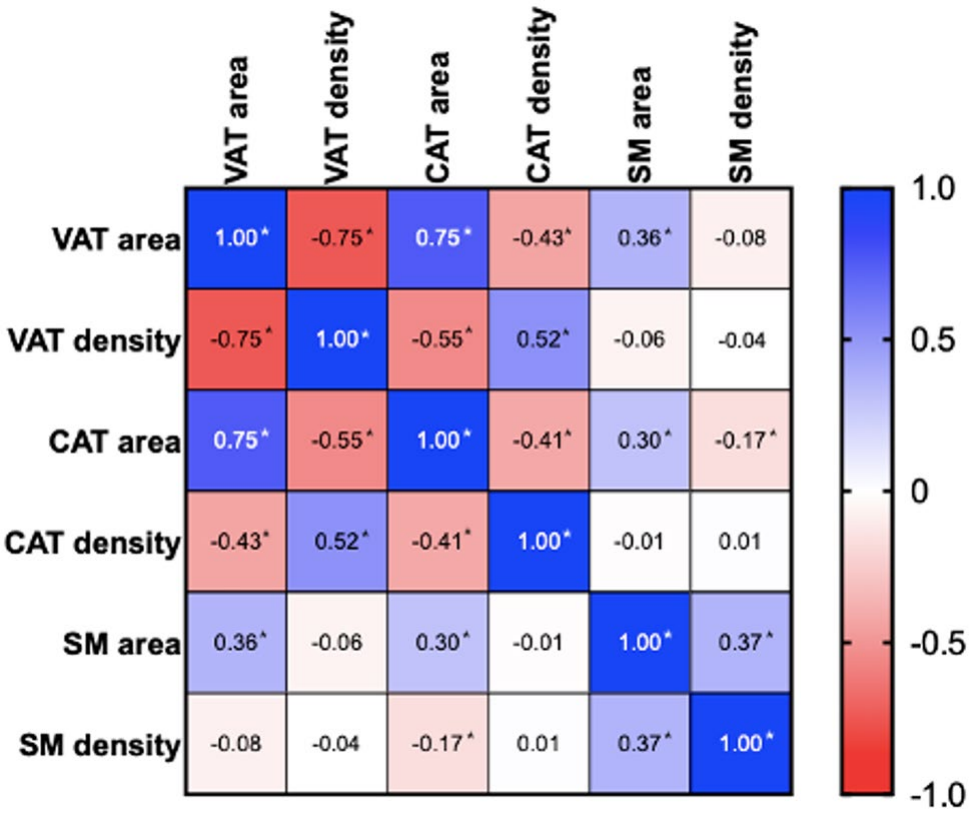
Heat map showing correlations between relevant CT-derived parameters of body composition in the study population (*n* = 842). CAT = cardiac adipose tissue, SM = skeletal muscle, VAT = visceral adipose tissue. Correlation coefficients marked with * indicate statistically significant values (*p* <.05 after correction).

In muscle tissue, SM area correlated moderately with VAT area (*r* =.364), CAT area (*r* =.300), and SM density (*r* =.366; all *P* <.001). The full correlation matrix is provided in Fig. 5.

### Sex-specific differences in anthropometric and CT-derived characteristics

Stratifying the cohort by sex revealed significant differences in age (79.5 ± 6.3 vs. 82.3 ± 5.8; *P* <.001), height (174.0 ± 6.8 vs. 140.7 ± 6.6 cm; *P* <.001) and weight (79.8 ± 14.5 vs. 68.4 ± 13.0 kg; *P* <.001) between men and women. However, BMI did not differ significantly between sexes (26.3 ± 4.4 vs. 26.3 ± 4.8 kg/m^2^; *P* <.001).

Also, notable disparities in abdominal body composition were evident. Men exhibited significantly higher VAT area (215.1 ± 115.4 vs. 140.7 ± 82.7 cm²; *P* <.001), SM area (146.7 ± 24.6 vs. 107.9 ± 18.4 cm²; *P* <.001), and CAT area (23.3 ± 11.7 vs. 17.3 ± 8.8 cm²; *P* <.001). Similarly, men had significantly greater EAT area (14.2 ± 6.8 vs. 11.7 ± 5.5 cm²; *P* <.001) and PAT area (9.1 ± 6.6 vs. 5.6 ± 4.6 cm²; *P* <.001). In contrast, men exhibited a significantly smaller SAT area compared to women (193.3 ± 89.9 vs. 228.9 ± 110.7 cm²; *P* <.001).

Regarding adipose tissue density, men had significantly lower CAT density (−86.3 ± 6.7 vs. −85.8 ± 6.1 HU; *P* <.001) and VAT density (−85.2 ± 11.1 vs. −82.9 ± 10.2 HU; *P* =.002) compared to women. A detailed comparison of the parameters is available in Table 2.

**Table 2:** Sex-specific anthropometric and CT characteristics of the study population (*n* = 842).

Variable	Male (*n* = 421)	Female (*n* = 421)	*P* value
*Anthropometric characteristics*			
Age (years)	79.5 ± 6.3	82.3 ± 5.8	**< 0.001**
Height (cm)	174.0 ± 6.7	161.1 ± 6.7	**< 0.001**
Weight (kg)	80.1 ± 14.7	68.3 ± 12.9	**< 0.001**
Body mass index (kg/m^2^)	26.3 ± 4.8	26.4 ± 4.6	0.577
*CT data*			
Visceral adipose tissue area (cm^2^)	215.1 ± 115.4	140.7 ± 82.7	**< 0.001**
Visceral adipose tissue density (HU)	−85.2 ± 11.1	−82.9 ± 10.2	**0.002**
Subcutaneous adipose tissue area (cm^2^)	193.3 ± 89.9	228.9 ± 110.7	**< 0.001**
Skeletal muscle tissue area (cm^2^)	146.7 ± 24.6	107.9 ± 18.4	**< 0.001**
Skeletal muscle tissue density (HU)	25.6 ± 7.7	20.9 ± 7.5	**< 0.001**
Cardiac adipose tissue area (cm^2^)	23.3 ± 11.7	17.3 ± 8.8	**< 0.001**
Cardiac adipose tissue density (HU)	−86.3 ± 6.7	−85.8 ± 6.1	0.126
Epicardial adipose tissue area (cm^2^)	14.2 ± 6.8	11.7 ± 5.5	**< 0.001**
Epicardial adipose tissue density (HU)	−80.5 ± 6.1	−82.0 ± 6.4	**< 0.001**
Paracardial adipose tissue area (cm^2^)	9.1 ± 6.6	5.6 ± 4.6	**< 0.001**
Paracardial adipose tissue density (HU)	−95.6 ± 9.8	−93.4 ± 8.9	**< 0.001**

**Fig. 6:**
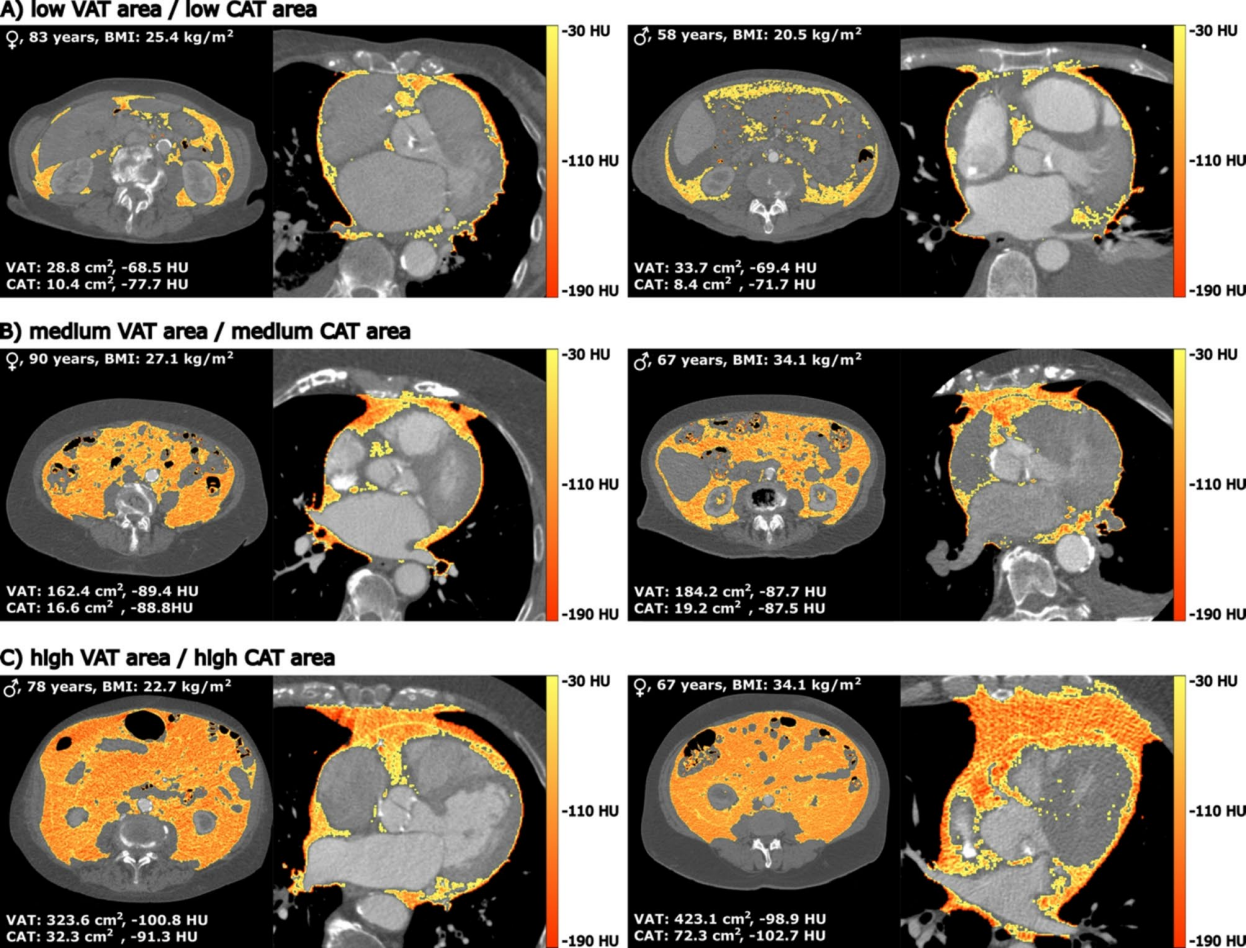
The images show exemplary male and female patients of different ages and BMI with low VAT and CAT, medium VAT and CAT, and high VAT and CAT. BMI = Body Mass Index, CAT = cardiac adipose tissue, HU = Hounsfield Units, SM = skeletal muscle, VAT = visceral adipose tissue.

Men also displayed stronger correlations between VAT density and CAT density (*r* =.620 vs. *r* =.403; *P* <.001). Correlations between VAT area, CAT area, and SM area did not differ significantly between sexes. Representative images of male and female patients of different ages with varying BMI, VAT, and CAT levels are presented in Fig. 6.

### Association between age and CT-derived characteristics

Multiple linear regression analyses revealed significant associations between age and several CT-derived body composition parameters. Advancing age was strongly associated with a decrease in VAT area (B = −256.1, *P* <.001) and CAT area (B = −25.8, *P* <.001). Also, VAT density showed a significant association with age (B = 0.26, *p* <.001), whereas CAT density did not exhibit a significant relationship (B = 0.03, *p* =.37). SM area declined significantly with age (B = −152.3, *P* <.001), and SM density also demonstrated a notable negative association (B = −0.38, *P* <.001) **(**Table 3**)**.

**Table 3:** Influence of age on relevant CT-derived parameters of body composition in the study population (*n* = 842).

Dependent variable	B (unstandardized)	SE (standard error)	Beta (standardized)	*P* value
Visceral adipose tissue area (cm^2^)	−256.1	59.0	0.15	**< 0.001**
Visceral adipose tissue density (HU)	0.26	0.1	0.14	**< 0.001**
Cardiac adipose tissue area (cm^2^)	−25.8	6.0	−0.15	**< 0.001**
Cardiac adipose tissue density (HU)	0.03	0.04	0.03	0.37
Skeletal muscle tissue area (cm^2^)	−152.3	15.4	−0.32	**< 0.001**
Skeletal muscle tissue density (HU)	−0.38	0.04	0.3	**< 0.001**

Stratifying the cohort by age revealed significantly stronger correlations in the younger group between SM area and VAT area (*r* =.400 vs. *r* =.267; *P* =.015) and between SM area and CAT area (*r* =.326 vs. *r* =.194; *P* =.02). In contrast, correlations between different adipose tissue parameters and within muscle parameters did not differ significantly between the groups.

### Association between BMI and CT-derived characteristics

Multiple linear regression analyses revealed significant associations between BMI and various CT-derived body composition parameters. Higher BMI was strongly associated with an increase in visceral adipose tissue (VAT) area (B = 1481.2, *P* <.001) and cardiac adipose tissue (CAT) area (B = 120.2, *P* <.001). In contrast, VAT density showed a significant negative association with BMI (B = −1.2, *P* <.001), as did CAT density (B = −0.3, *P* <.001). Skeletal muscle (SM) area increased significantly with BMI (B = 183.2, *P* <.001), whereas SM density exhibited a significant negative association (B = −0.25, *P* <.001) **(**Table 4**)**.

**Table 4:** Influence of body mass index on relevant CT-derived parameters of body composition in the study population (*n* = 842).

Dependent variable	B (unstandardized)	SE (standard error)	Beta (standardized)	*P* value
Visceral adipose tissue area (cm^2^)	1481.2	64.1	0.65	**< 0.001**
Visceral adipose tissue density (HU)	−1.2	0.01	−0.53	**< 0.001**
Cardiac adipose tissue area (cm^2^)	120.2	7.3	0.52	**< 0.001**
Cardiac adipose tissue density (HU)	−0.3	0.05	−0.23	**< 0.001**
Skeletal muscle tissue area (cm^2^)	183.2	22.0	0.29	**< 0.001**
Skeletal muscle tissue density (HU)	−0.25	0.06	−0.14	**< 0.001**

When stratifying the cohort by BMI, the lower BMI group showed significantly weaker correlations between VAT area and SM area (*r* =.125 vs. *r* =.442; *P* <.001), whereas correlations between VAT area and VAT density were significantly stronger (*r* = -.721 vs. *r* = -.636; *P* =.011). No significant differences were observed between groups in the correlations between VAT and CAT area or VAT and CAT density.

## Discussion

In the present study, we investigated potential associations between CT-derived parameters of CAT and abdominal muscle and adipose tissue composition, established as surrogates for the patients general condition. Strong associations were observed, particularly between the area and density values of visceral and cardiac adipose tissue compartments. Moderate associations were also detected among the composition parameters of abdominal skeletal muscle tissue and the different adipose tissue compartments. These associations indicate a systematic pattern in changes to body composition, advocating for a comprehensive evaluation of body composition in future compositional analyses.

With advancing age, body composition undergoes structural changes^24^. These changes, among other factors, are attributed to an age-related increase in pro-inflammatory processes in the body, often referred to as “inflammaging”^4,5,25,26^. In this context, adipose tissue as the largest endocrine organ in humans plays a central role^6^. Inflammatory processes associated with inflammaging also lead to structural changes in adipose tissue composition^27,28^. It is hypothesized that with aging, adipose tissue becomes increasingly dysfunctional, affecting the differentiation of preadipocytes into mature adipocytes^27^. This growing dysfunction is often attributed to heightened activation of immune cells infiltrating the adipose tissue, producing cytokines and chemokines, resulting in persistent chronic inflammation, thereby impairing local adipogenesis^29^. Dysfunctional adipogenesis triggered by inflammatory processes results in a shift of density values from the lipid phase to the aqueous phase^21^, which can be quantified through CT imaging. Correlations between laboratory-measured inflammatory activity and adipose tissue density, have been demonstrated in various studies across different fat depots, including CAT and VAT^21,30,31^. These studies advocate for the utilization of adipose tissue density as a biomarker for inflammatory activity, including CAT density^3,30,32^.

Importantly, inflammatory changes in CAT are not solely attributable to age-related alterations. Rather, these chronic, low-grade inflammatory processes can be influenced by metabolic changes as seen in obesity (“metaflammation”) or genetic factors^33–35^. Therefore, it is crucial to consider CT-derived adipose tissue density not as a parameter for inflammaging but rather as a surrogate marker for inflammatory adipose tissue activity and the patient’s clinical status.

Our study revealed strong correlations between density changes in CAT and VAT, independent of sex, age, or BMI group. These presented findings not only indicate that alterations in adipose tissue composition occur in different compartments but also suggest interrelationships among changes in the various adipose tissue compartments. This observation supports the hypothesis that inflammatory processes do not only affect the body regionally but rather systemically, with associated changes interlinked across different adipose tissue compartments.

Studies also suggest that disrupted adipogenesis due to inflammatory processes leads to a subsequent reduction in adipose tissue volume, as undifferentiated adipocytes become less capable of storing fat^27^. Supporting this hypothesis, our study cohort exhibited moderate to strong negative correlations between the areas and density values of various adipose tissue compartments.

Furthermore, in our study patients with lower adipose tissue area also displayed lower skeletal muscle area. Here, again underlying inflammatory processes serve as potential explanations. Regarding biological mechanisms, the development and progression of frailty are frequently associated with a systemic inflammatory state^10,25^. While connections between physical frailty and inflammatory processes are not yet fully understood, they are well-documented in aging literature^25^. In this context, the robust correlations observed in our study between muscle and adipose tissue compartments also suggest a potential systematic relationship between structural changes in adipose and muscle composition, warranting a holistic approach in future studies analyzing body composition and necessitating further investigations in this direction.

Over the past few years, there has been an increasing focus on opportunistic assessment of body composition parameters using cross-sectional images, as this approach has been shown to offer important predictive insights in patients with various malignancies and cardiometabolic disorders. Particularly, VAT area and density have been shown to have prognostic value for the outcome of numerous malignancies and cardiovascular diseases^1,8,14,15,36^. Recently, quantitative and qualitative changes in CAT have come into focus as specific prognostic biomarkers for the outcome of cardiovascular disease and cardiac-specific interventions^3,15,30,37,38^. Whether characteristics of CAT can be used as prognostic biomarkers for the outcome of non-cardiovascular disease has not yet been investigated. Our study uncovered strong associations between the features of abdominal and cardiac adipose tissue compartments, suggesting that changes in CAT composition might also offer valuable prognostic insights into non-cardiovascular disease outcomes. If included along with well-established body composition parameters with predictive value for non-cardiovascular disease outcomes, CAT features may have the potential to improve outcome predictions across diverse diseases. These implications warrant further investigation in future studies.

We acknowledge several limitations in our study, including its retrospective design and the advanced age of the study participants. Additionally, a certain number of patients were excluded from the analysis due to the unavailability of suitable images. For instance, images were deemed unsuitable when precise segmentation was impeded by artifacts caused by metallic implants in the coronary arteries. Another limitation arises from the use of arterial phase contrast-enhanced CT scans, which may affect tissue density measurements. This could introduce variability when comparing tissue densities across individuals—for example, individuals with lower skeletal muscle mass may exhibit relatively higher density values due to greater local contrast concentration. Although image acquisition was highly standardized, this potential confounding effect should be considered when interpreting tissue density values.

Importantly, the primary objective of this study was to investigate the associations between parameters of abdominal and cardiac adipose tissue and muscle composition. These parameters are recognized for their well-documented high metabolic activity and demonstrated prognostic relevance in various pathologies, serving as surrogates for the clinical status of patients. However, we cannot fully exclude the possibility that the observed associations—for instance between lower adipose tissue and skeletal muscle area—are at least partly influenced by overall body size rather than solely reflecting underlying pathophysiological mechanisms. This should be addressed more systematically in future investigations. Future studies should also explore the associations with other prognostically relevant parameters of body composition, such as bone density or vascular calcification^39,40^. Nevertheless, the demonstrated strong associations between parameters of cardiac adipose tissue and abdominal muscle and adipose tissue composition underscore the robustness of the observed systematic relationships.

In conclusion, this study investigated associations between the composition of cardiac adipose tissue and abdominal adipose and muscle tissue, indicating a systematic pattern of interrelated changes across these compartments. These findings underscore the interconnected nature of body composition alterations, likely driven by shared underlying systemic processes such as chronic inflammation and metabolic dysregulation. The observed relationships advocate for the inclusion of comprehensive body composition analysis in future studies to deepen our understanding of their clinical implications across diverse pathological contexts.

To account for alpha errors due to multiple testing, the Bonferroni correction was applied. 

## Data Availability

Data generated or analyzed during the study are available from the corresponding author by reasonable request.
